# Simultaneous Transcatheter Pulmonary and Tricuspid Valve Replacement in Carcinoid Heart Disease

**DOI:** 10.1016/j.jaccas.2025.103546

**Published:** 2025-03-26

**Authors:** Varius Dannenberg, Caglayan Demirel, Markus Raderer, Barbara Kiesewetter, Lukas Reider, Anna Bartunek, Martin Andreas, Daniel Zimpfer, Jutta Bergler-Klein, Christian Hengstenberg, Philipp E. Bartko

**Affiliations:** a Department for Internal Medicine II, Clinical Division of Cardiology, Medical University of Vienna, Vienna, Austria; b Department of Internal Medicine I, Clinical Division of Oncology, Medical University of Vienna, Vienna, Austria; c Department of Interventional Radiology, Medical University of Vienna, Vienna, Austria; d Department of Anaesthesiology, General Intensive Care and Pain Medicine, Division of Cardiac Thoracic Vascular Anesthesia and Intensive Care Medicine, Medical University Vienna, Vienna, Austria; e Department of Cardiac Surgery, Medical University of Vienna, Vienna, Austria

**Keywords:** carcinoid heart disease, echocardiography, neuroendocrine tumor, pulmonary valve, tricuspid valve, valve replacement

## Abstract

**Background:**

Neuroendocrine tumors can cause carcinoid heart disease, often presenting with pulmonary and tricuspid regurgitation. Valvular pathology and right heart failure can influence prognosis more than the tumor itself. Given high surgical risk, prolonged recovery, and limited life expectancy, interventional valve replacement is a strong alternative.

**Case Summary:**

We present a patient with severe pulmonary and torrential tricuspid regurgitation caused by carcinoid heart disease. Despite stable neuroendocrine tumor control, the patient developed progressive right heart failure, requiring urgent valve therapy. Simultaneous transcatheter pulmonary and tricuspid valve replacements were performed, almost eliminating tricuspid and pulmonary regurgitation.

**Discussion:**

Right heart valve failure is frequent in carcinoid heart disease and significantly impacts outcomes. Although surgical valve replacement remains the standard, transcatheter approaches provide an effective, less invasive alternative for high-risk patients, offering symptom relief and excellent results.

**Take-Home Message:**

Simultaneous interventional valve replacement in carcinoid heart disease is feasible and effective.

## Visual Summary

**Figure:**
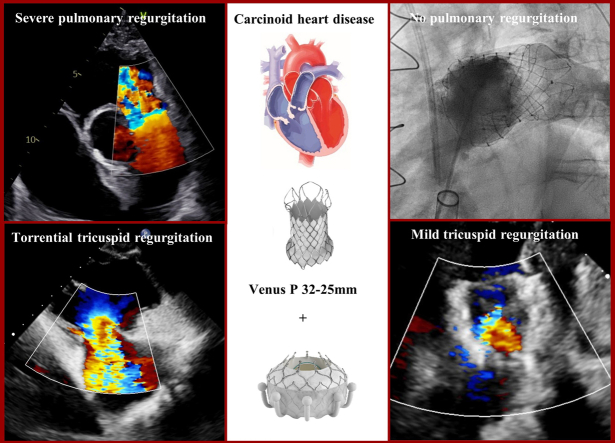

Carcinoid heart disease (CHD) is a degenerative condition of the heart valves caused by substances, particularly serotonin, released by neuroendocrine tumors (NETs).^1^ Carcinoid tumors occur in 1 to 5 per 100,000 people, most often in the gastrointestinal tract, with the midgut being the primary site for functional disease.^2^, ^3^, ^4^ Treatment depends on the tumor’s location, grading, and symptoms and includes somatostatin analogs, rapamycin inhibitors, tyrosine kinase inhibitors, interferon alfa, and cytostatic agents.^5^

**Take-Home Messages**

• Patients with NET and CHD are at high risk for right heart valve degeneration, resulting in severe pulmonary and tricuspid regurgitation.
• Interventional replacement of both valves is possible in a simultaneous procedure, providing effective therapy for patients with high surgical risk.

In CHD, gastrointestinal tumors release vasoactive substances like serotonin into the portal circulation, where they are metabolized in the liver. However, liver metastases bypass this process, allowing these substances to reach the right heart, where they are typically eliminated in the lungs, preventing left heart involvement. Consequently, right heart valve degeneration occurs in over 90% of cases.^6^^,^^7^ Excess serotonin promotes fibrosis by stimulating cardiac fibroblast growth, forming plaque on valve leaflets.^4^

Over 95% of patients with valvular involvement have tricuspid valve disease, with 90% experiencing significant tricuspid regurgitation (TR). The pulmonary valve is affected in 85% of cases, presenting as pulmonary regurgitation (PR) in 80% and pulmonary stenosis in 50%.^8^ Valve replacement is typically recommended following current valvular heart disease guidelines.^9^ Although surgical valve replacement remains the standard, transcatheter approaches are emerging for high-risk patients, primarily for degenerated prostheses.^10^ In native anatomy, the Venus P-Valve (Venus Medtech Inc) is available for transcatheter pulmonary valve replacement (TPVR),^11^ and the EVOQUE system (Edwards Lifesciences) for transcatheter tricuspid valve replacement (TTVR) has been shown to provide substantial clinical improvement.^12^ Successful Venus P-Valve implantations in patients with CHD have recently been published.^13^

We report a CHD patient with severe PR and torrential TR in native anatomy, treated with a Venus P-Valve and an EVOQUE in a single procedure.

## Case Presentation

### History of presentation

We present a 66-year-old man with severe, progressive dyspnea over the last few weeks who was referred from the oncology department.

### Past medical history

Three years ago, the patient was diagnosed with a primary metastasized midgut NET with a low proliferation rate (G1, World Health Organization classification). He initially presented with elevated inflammatory parameters and unexplained weight loss. Elevated urinary 5-Hydroxyindoleacetic acid levels confirmed functional activity. Since diagnosis, he has been treated with long-acting release octreotide. One year later, he underwent ileum and coecum resection. Liver metastases (<5 cm) remained stable, and oncologists rated his long-term prognosis as good. Regarding his cardiac history, he received a left internal mammary artery (LIMA) bypass to the left anterior descending (LAD) coronary artery 26 years ago. Shortly after the operation, the LIMA occluded at the ostium, necessitating acute redo surgery, where a venous bypass graft was connected to the LIMA. This ensured perfusion of the LAD via the vein graft and the distal LIMA. The native LAD originated atypically from the right coronary cusp, with severe but incomplete ostial stenosis. Significant stenoses were also found proximally and distally in the right coronary artery, and borderline stenosis was found at the circumflex ostium. These were treated conservatively to avoid triple anticoagulation. Future symptoms were to be managed with intervention if necessary. The patient also suffered from peripheral artery disease, which required an aortofemoral bypass 23 years ago, and he has insulin-dependent type II diabetes.

### Investigations

**Figure 1:**
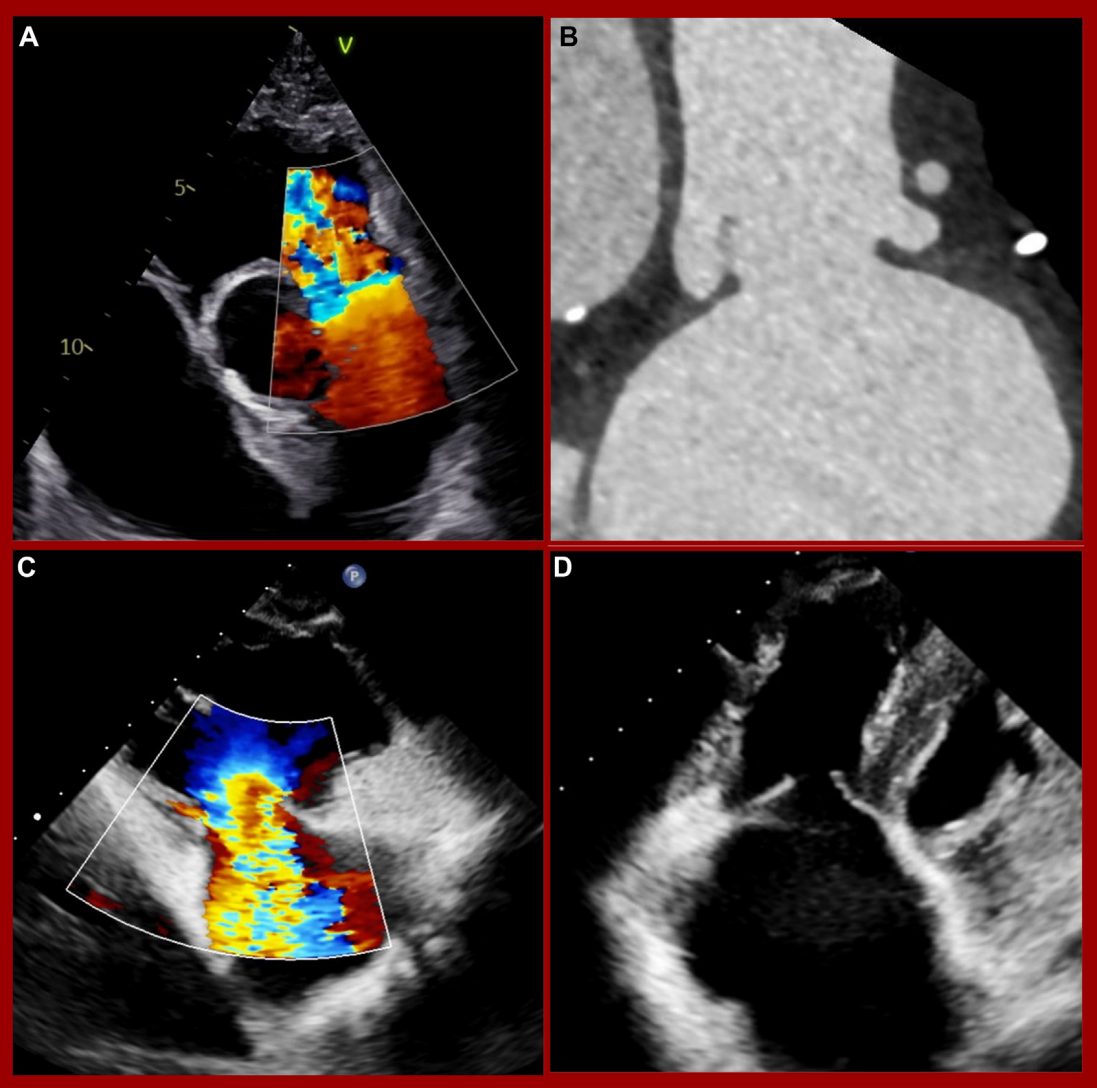
Baseline Pulmonary and Tricuspid Regurgitation (A) Severe pulmonary regurgitation. (B) Destructed pulmonary valve. (C) Torrential tricuspid regurgitation. (D) Large coaptation gap of the tricuspid valve.

Electrocardiogram revealed sinus rhythm with a first-degree atrioventricular block (205 milliseconds) and normal QRS duration (102 milliseconds). Transthoracic echocardiography showed torrential TR and severe PR caused by degenerated and thickened leaflets (Figures 1A and 1B), with both lesions worsening compared with earlier evaluations. Mild-to-moderate regurgitation of the aortic and mitral valves was also present. The left ventricle was normal in size, the right ventricle was enlarged, and both ventricular functions were preserved. Hemodynamics indicated mild pulmonary hypertension with elevated right atrial pressure. Transesophageal echocardiography confirmed these findings and demonstrated good esophageal and transgastric image quality. All baseline characteristics are summarized in Table 1.

**Table 1:** Patient Details

Baseline characteristics	
Sex	Male
Age	66 y
Dyspnea	NYHA functional class II-III
ECG	Sinus rhythm, QRS duration 102 milliseconds, AVB first degree (205 milliseconds)
Coronary angiography	Atypical LAD origin from right coronary cusp LIMA bypass to LAD, vein bypass to LIMA LIMA ostium occluded, proximal LAD occluded, vein bypass with good flow Significant proximal and distal RCA stenosis (conservative management) Borderline stenosis of the proximal CX
Renal function	Creatine: 0.88 mg/dL
NT-proBNP	1,508 pg/mL
Preexisting conditions	Neuroendocrine tumor for >3 y Carcinoid heart disease Hepatic metastases of at most 5 cm Favorable long-term prognosis History of ileocecal resection October 2022 Coronary artery disease with CABG September 1998 PAD with aortofemoral bypass 2001 Insulin-dependent diabetes mellitus
Medication	Ramipril, amlodipine, bisoprolol, empagliflozin, finerenone, atorvastatin, pantoprazole, octreotide
EuroScore-II	14.36%
Invasive hemodynamics	
Pulmonary artery pressure (systolic/diastolic/mean)	37/13/20 mm Hg
Pulmonary capillary wedge pressure (atrial/ventricular/mean)	14/16/13, mm Hg
Right atrial pressure (atrial/ventricular/mean)	15/30/16 mm Hg
Echocardiography	
Tricuspid valve	
Regurgitation, vena contracta, EROA	Torrential, 21 mm, 0.9 cm^2^
Stenosis, vmax, mean gradient	Not relevant, 1.2 m/s, 3 mm Hg
Pulmonary valve	
Regurgitation, vena contracta, EROA	Severe, 25 mm, NA
Stenosis, vmax, mean gradient	Not relevant, 1.3 m/s, 2 mm Hg
Right ventricular size and function	Dilated and preserved
Right ventricular end-diastolic diameter	59 mm
TAPSE, TDI s', FAC	21 mm, 12 cm/s, 0.5
Left ventricular size and function	Normal and preserved
Left ventricular end-diastolic volume	87 mL
Ejection fraction	62%
(Post) interventional details	
Access	Right femoral vein, 27-F
Prosthesis pulmonary	Venus P Valve 32-25 mm
Prosthesis tricuspid	EVOQUE 44 mm
Pulmonary regurgitation	None
Tricuspid regurgitation	Mild
Procedural time, min	186
ECG	Sinus rhythm, QRS duration 138 milliseconds, complete RBBB
Anticoagulation	Edoxaban
Mean tricuspid inflow gradient	3 mm Hg
Mean pulmonary outflow gradient	1 mm Hg

AVB = atrioventricular block; CABG = coronary artery bypass graft; CX = circumflex artery; ECG = electrocardiogram; EROA = effective regurgitation orifice area; FAC = fractional area change; LAD = left anterior descending; LIMA = left internal mammary artery; NA = not applicable; NT-proBNP = N-terminal pro–B-type natriuretic peptide; PAD = periphery artery disease; RBBB = right bundle branch block; RCA = right coronary artery; TAPSE = tricuspid annulus plane systolic excursion; TDI = tissue Doppler imaging; vmax = maximal velocity.

### Management

**Figure 2:**
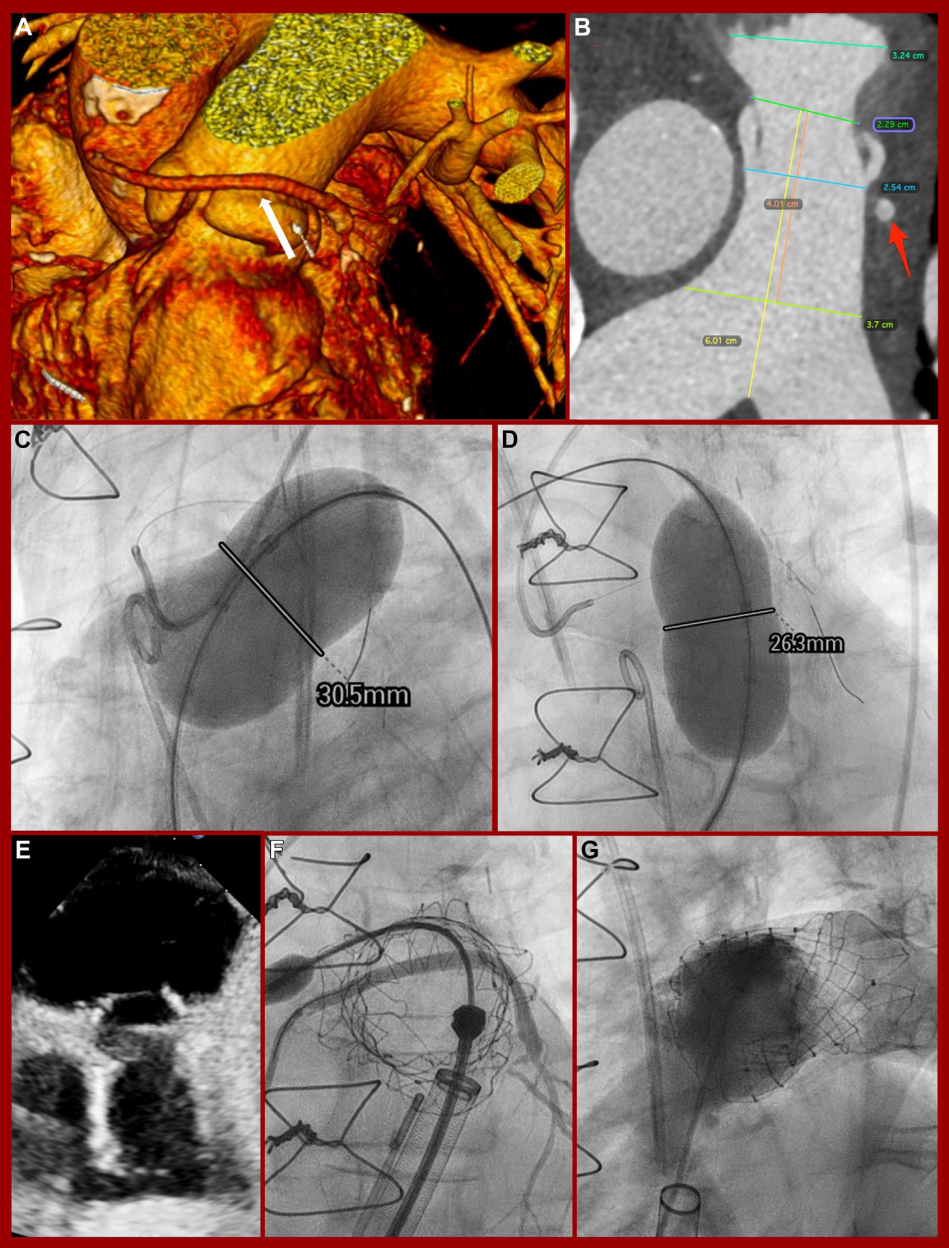
Sizing, Implantation, and Procedural Results of Venus P Valve (A) Coronary venous bypass graft (white arrow). (B) Computed tomography sizing of the main pulmonary artery (red arrow = coronary venous bypass graft). (C and D) Balloon sizing of the main pulmonary artery. (E) Implanted Venus P-Valve. (F) Down-the-barrel view with venous bypass graft contrast. (G) No pulmonary regurgitation in contrast fluoroscopy.

**Figure 3:**
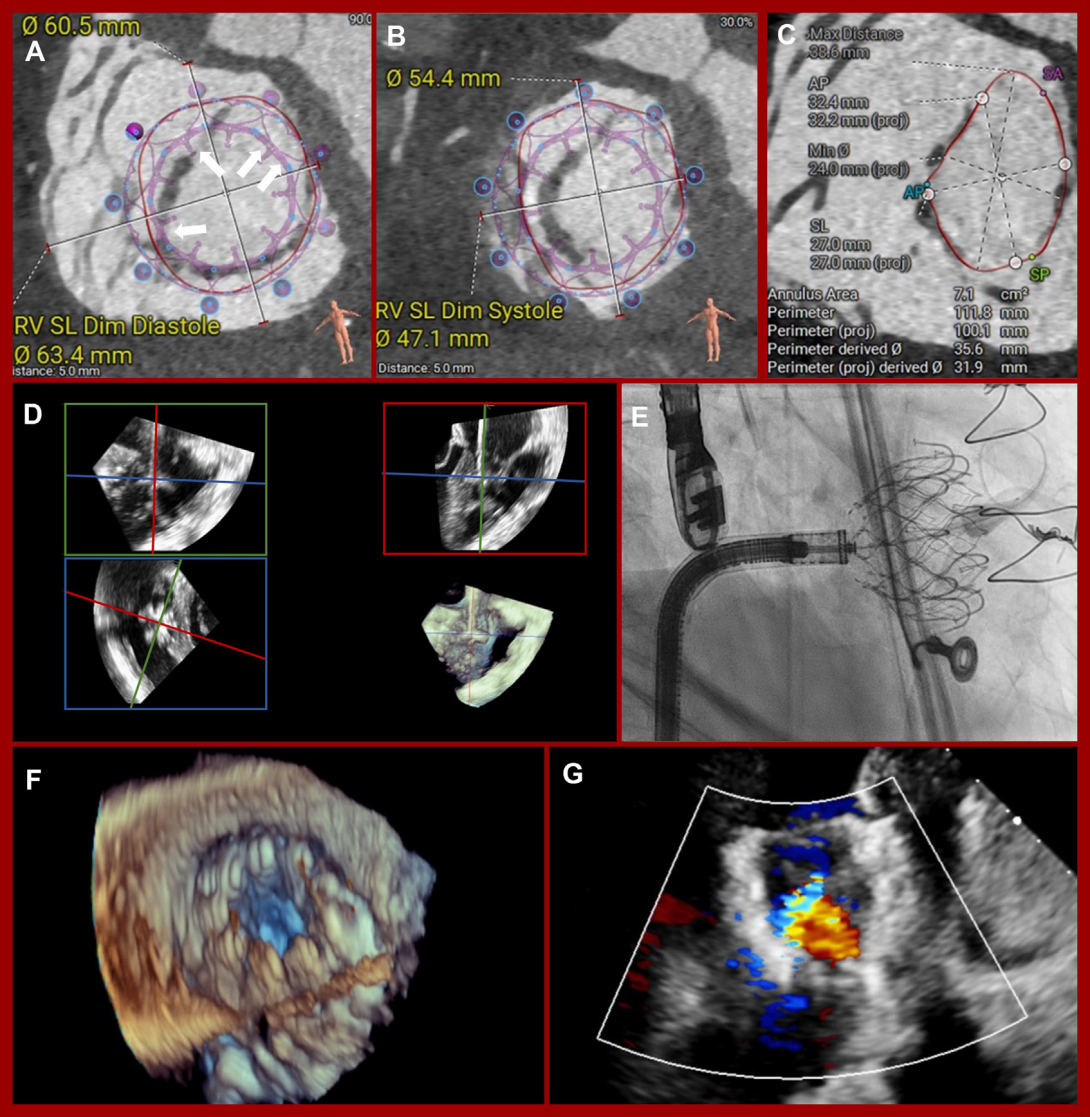
Sizing, Implantation, and Procedural Results of EVOQU (A) Computed tomography sizing of the tricuspid anatomy in diastole. Interruption of the thickened and fused leaflets (open arrows). (B) Computed tomography sizing of the tricuspid anatomy in systole. (C) Tricuspid valve annulus at leaflet tip level. (D) Implantation of the EVOQUE using multiplane reconstruction. (E) Full atrial deployment. (F) Stable postinterventional position. (G) Mild valvular tricuspid regurgitation.

The patient underwent a computed tomography (CT) scan to evaluate suitability for TPVR and TTVR. CT confirmed restricted, thickened valve leaflets with limited mobility (Figures 1C and 1D). A prestenting of the pulmonary artery using the Alterra Adaptive Prestent System (Edwards Lifesciences) was considered to protect the venous bypass graft, but the product was unavailable at that time. The creation of a landing zone with bare stents to implant a balloon-expandable valve in a second step was also considered but rejected due to the healing phase and the associated delay in therapy for a highly symptomatic patient. Therefore, a Venus P-Valve 32-25 mm (Venus Medtech Inc) for TPVR and a 44 mm EVOQUE (Edwards Lifesciences) for TTVR seemed the best treatment option. The heart team at the Medical University of Vienna determined that treatment of both valves was the best option for this patient, aligning with current cardio-oncology guidelines.^14^ Due to the patient’s high surgical risk, an interventional strategy was preferred. However, according to the guidelines for congenital heart disease,^15^ an isolated procedure on the tricuspid valve with close monitoring of the right ventricular volume using magnetic resonance imaging would also have been a feasible treatment option. The procedure was performed under general anesthesia with intubation and invasive hemodynamic monitoring. The anesthesia team used low-dose dobutamine and levosimendan for inotropic and vasopressor support, supplemented with low-to-moderate doses of noradrenaline as needed. The interventional team began with TPVR, accessing the femoral vein under ultrasound guidance and placing 2 suture-based vascular closure systems (Perclose ProStyle; Abbott). Preprocedural CT showed the proximity of the venous bypass graft to the valve implantation plane (Figure 2A). Balloon sizing of the main pulmonary artery confirmed the choice of a Venus P-Valve 32 mm in diameter and 25 mm in length (Figure 2B). A wire (Asahi Sion blue ES, ASAHI INTECC CO, LTD) was placed near the bypass graft for immediate stenting if needed. Balloon sizing confirmed graft patency during inflation and the suitability of the prosthesis (Figures 2C and 2D). The Venus P-Valve was successfully delivered via a 26-F Gore Dryseal introducer (Gore Medical) and deployed at the origin of the right pulmonary artery. Postprocedural angiography confirmed bypass graft patency, and echocardiography showed excellent prosthesis fitting (Figures 2E to 2G) with no PR. After TPVR, the team proceeded to TTVR. CT screening identified a 44 mm EVOQUE bioprosthesis as suitable for implantation (Figures 3A and 3B). Thickened leaflet tips, partially fused (Figure 2C), were a focus of concern. The 28-F EVOQUE delivery system was guided using multiplane reconstruction imaging and advanced into the right atrium and ventricle (Figure 2D). Before prosthesis deployment, a preshaped stiff guidewire (Safari XS; Boston Scientific Corp) was positioned in the right ventricular apex using a steerable sheath (Agilis NxT; Abbott). The delivery system was oriented perpendicular to the tricuspid annulus, and the prosthesis was gradually released into its final position (Figure 2E). Postinterventional echocardiography showed good valve positioning with mild valvular regurgitation (Figures 2F and 2G), trivial paravalvular regurgitation, and a mean transvalvular inflow gradient of 3 mm Hg. The system was successfully retrieved, and femoral vein access was closed with Perclose ProStyle sutures. The procedure lasted 186 minutes, with successful extubation and transfer to the intensive care unit for monitoring. Catecholamine support was gradually tapered, and the patient was moved to the general ward the following day.

### Outcome

Postinterventional transthoracic echocardiography confirmed excellent valve function. The Venus P-Valve had a mean transpulmonary outflow gradient of 1 mm Hg with no PR, whereas the EVOQUE showed mild TR with a mean inflow gradient of 3 mm Hg. The patient developed a right bundle branch block (QRS prolongation to 138 milliseconds, +36 milliseconds) but had no further conduction disturbances. There were no access site complications. Slightly elevated inflammatory markers were treated with intravenous antibiotics, and anticoagulation with edoxaban was initiated. The patient was discharged in good condition and scheduled for close follow-up care.

## Discussion

Here, we present a patient with NET leading to severe PR and torrential TR who was successfully treated with TPVR and TTVR within 1 procedure, resulting in almost complete elimination of both valvular regurgitations.

NETs are typically diagnosed in the fourth or fifth decade of life, with CHD often following between 50 and 70 years of age.^1^^,^^16^ These relatively young patients are usually treated surgically. Our patient, however, had early-onset diabetes mellitus and coronary artery disease by 40 years of age, requiring bypass surgery. A second surgery followed due to bypass occlusion, raising his perioperative risk to a EuroScore II of 14.36%.^17^

The feasibility of combined TPVR and TTVR has been demonstrated in valve-in-valve procedures using the Sapien S3 and Sapien XT (Edwards Lifesciences) for degenerated prostheses and native anatomy.^18^ For the first time, we showed the feasibility of TPVR and TTVR in native anatomy using the EVOQUE and Venus P-Valve for CHD. We selected a smaller Venus P-Valve size to avoid coronary and bypass compression, accepting a slightly higher embolization risk. The postprocedural transvalvular gradient was 1 mm Hg, with no signs of bypass compression.

During EVOQUE screening, the thickened and fused leaflets appeared to form a ring at the leaflet tips, potentially making valve expansion difficult. However, this ring-like structure was incomplete, with interruptions posteriorly, septally, and laterally (Figure 3A, open arrows). We chose the smallest prosthesis (44 mm) to minimize migration risk, with larger valves expanding against resistance. In our case, slight migration toward the ventricle occurred after atrial deployment, but the final result was excellent, with only minimal valvular regurgitation.

Anticoagulation strategies remain uncertain, given the thrombosis risk of prosthetic valves in CHD.^7^ Based on bleeding risks with vitamin K antagonists,^19^ we used direct oral anticoagulants with close follow-up.

Patients with serotonin-producing NETs face high morbidity and mortality driven by CHD. Timely surgical or interventional valve replacement should not be delayed for stable oncologic disease, highlighting the need for an interdisciplinary cardio-oncology team.^20^, ^21^, ^22^, ^23^

## Conclusions

We report the first successful use of a Venus P-Valve and EVOQUE prosthesis in a combined procedure for a 66-year-old NET patient with CHD, severe PR, and torrential TR. Both regurgitations were almost eliminated.

**Visual Summary:**
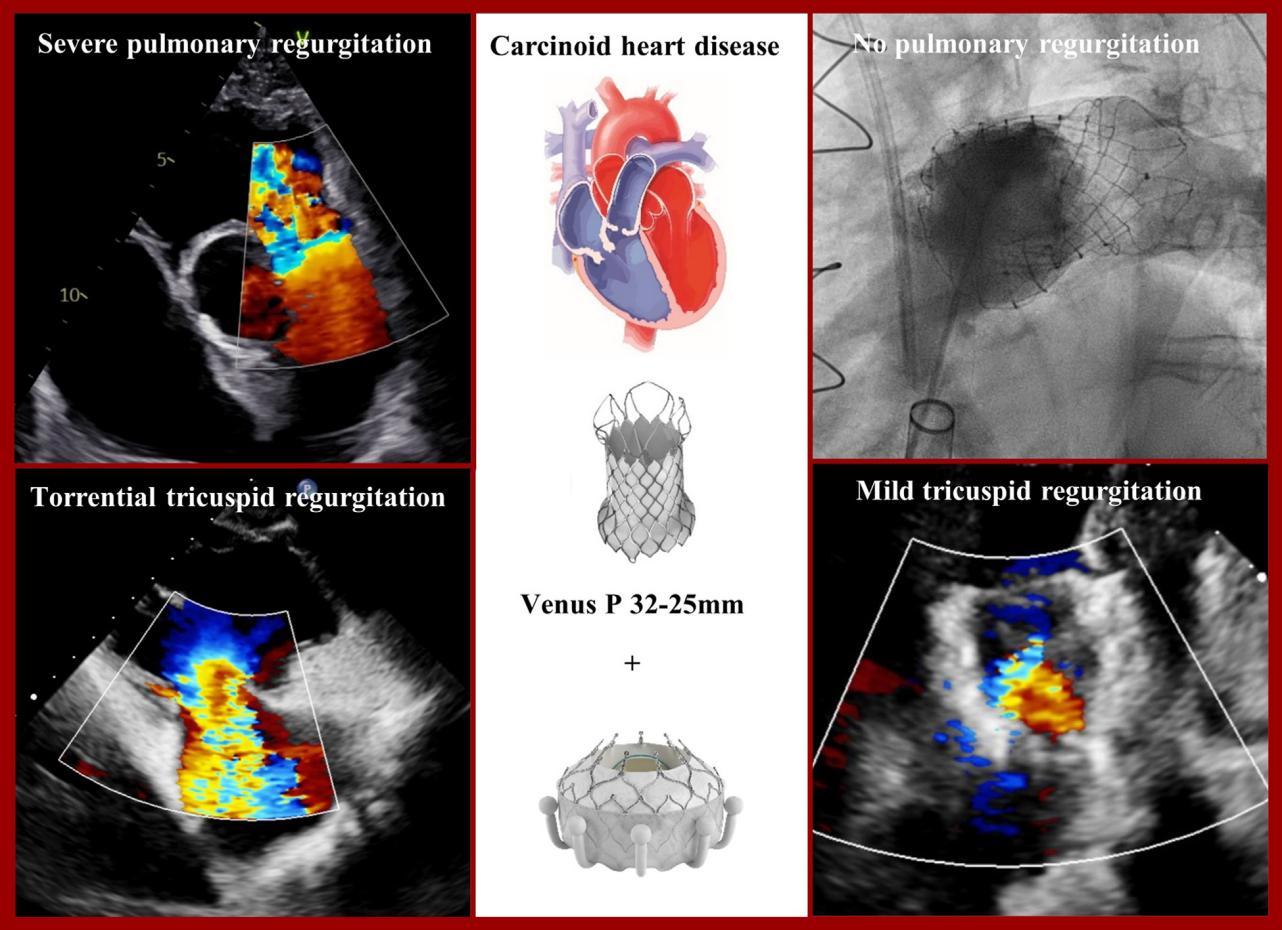
Simultaneous Transcatheter Pulmonary and Tricuspid Valve Replacement in Carcinoid Heart Disease

## Funding Support and Author Disclosures

Dr Dannenberg has been a proctor/speaker for Abbott and Edwards Lifesciences. Dr Bartko has been a proctor/speaker for Abbott and Edwards Lifesciences. Dr Andreas has been a proctor/consultant/speaker for Edwards Lifesciences, Abbott, Medtronic, Boston, B. Braun, and Zoll; and reports institutional research grants from Edwards, Abbott, Medtronic, and LSI. All other authors have reported that they have no relationships relevant to the contents of this paper to disclose.
